# Role of Oxidative Stress Signaling, Nrf2, on Survival and Stemness of Human Adipose-Derived Stem Cells Exposed to X-rays, Protons and Carbon Ions

**DOI:** 10.3390/antiox13091035

**Published:** 2024-08-26

**Authors:** Mira Hammad, Rima Salma, Jacques Balosso, Mohi Rezvani, Siamak Haghdoost

**Affiliations:** 1 Centre de Recherche sur les Ions, les Matériaux et la Photonique (CIMAP) UMR 6252, University of Caen Normandy, Cedex 04, F-14050 Caen, France; 2 Department of Radiation Oncology, Centre François Baclesse, F-14000 Caen, France; 3 Advanced Resource Center for HADrontherapy in Europe (ARCHADE), F-14000 Caen, France; 4 Swiss Bioscience GmbH, Wagistrasse 27a, CH-8952 Schlieren, Switzerland; mrezvani@btinternet.com; 5 Le Laboratoire “Aliments, Bioprocédés, Toxicologie et Environnement (ABTE) UR 4651, ToxEMAC Team, University of Caen Normandy, Cedex 04, F-14050 Caen, France; 6 Department of Molecular Biosciences, The Wenner-Gren Institute, Stockholm University, SE-10691 Stockholm, Sweden

**Keywords:** adipose-derived stem cell, ADSC, Nrf2 inhibitor, X-rays, protons, carbon ions, ionizing radiation, osteogenesis, adipogenesis, differentiation, radiotherapy, particle radiation

## Abstract

Some cancers have a poor prognosis and often lead to local recurrence because they are resistant to available treatments, e.g., glioblastoma. Attempts have been made to increase the sensitivity of resistant tumors by targeting pathways involved in the resistance and combining it, for example, with radiotherapy (RT). We have previously reported that treating glioblastoma stem cells with an Nrf2 inhibitor increases their radiosensitivity. Unfortunately, the application of drugs can also affect normal cells. In the present study, we aim to investigate the role of the Nrf2 pathway in the survival and differentiation of normal human adipose-derived stem cells (ADSCs) exposed to radiation. We treated ADSCs with an Nrf2 inhibitor and then exposed them to X-rays, protons or carbon ions. All three radiation qualities are used to treat cancer. The survival and differentiation abilities of the surviving ADSCs were studied. We found that the enhancing effect of Nrf2 inhibition on cell survival levels was radiation-quality-dependent (X-rays > proton > carbon ions). Furthermore, our results indicate that Nrf2 inhibition reduces stem cell differentiation by 35% and 28% for adipogenesis and osteogenesis, respectively, using all applied radiation qualities. Interestingly, the results show that the cells that survive proton and carbon ion irradiations have an increased ability, compared with X-rays, to differentiate into osteogenesis and adipogenesis lineages. Therefore, we can conclude that the use of carbon ions or protons can affect the stemness of irradiated ADSCs at lower levels than X-rays and is thus more beneficial for long-time cancer survivors, such as pediatric patients.

## 1. Introduction

Ionizing radiation is widely used in the treatment of cancer because of its ability to eradicate malignant cells. However, during radiation therapy (RT), healthy normal tissues in the vicinity of the tumor and those in the radiation path are also affected. Damage to normal healthy tissue by ionizing radiation is the most limiting side effect of radiotherapy. As well as being a side effect of radiotherapy, radiation damage to normal tissues is a serious health hazard for populations exposed to ionizing radiation accidentally or because of nuclear warfare. Side effects associated with radiotherapy can manifest during the course of therapy, a few weeks, months or years after radiotherapy [1,2], leading to undesirable healthy tissue effects, e.g., cardiac diseases, neuro/cardiovascular diseases, radiation pneumonitis, nephritis, fibrosis, second primary cancer and necrotic soft tissues and bones (osteoradionecrosis).

Radiation exerts its detrimental effects on cells through both direct and indirect effects. The direct effect occurs when radiation interacts directly with cellular components, including DNA, proteins and lipids, and modifies their structures. The indirect effect of ionizing radiation refers to the interaction of radiation with intracellular water, leading to radiolysis products such as reactive oxygen species (ROS). ROS can then react with, e.g., DNA, proteins and lipids, and alter their structures, ultimately impairing their normal physiological functions and triggering various effects in cells [3]. Furthermore, radiation-induced ROS can trigger a cascade of inflammatory responses, affecting nearby cells and propagating tissue damage [4]. Molecular events result in the loss of both stem cells and differentiated functional cells in that tissue. At the molecular level, increased levels of ROS also trigger several signaling pathways that contribute to the increased expression of proteins involved, for example, in the cellular antioxidant system or DNA repair mechanism, leading to improved survival [5].

An essential response pathway that is activated when bodily tissue is faced with high levels of ROS (oxidative stress condition) involves the activation of the nuclear factor NF-E2-related factor 2 (Nrf2) pathway. The Nrf2 pathway regulates cellular defense against oxidative stress. Nrf2 is also recognized as a master regulator of endogenously [6] or exogenously induced oxidative stress, such as during exposure to low as well as to high linear energy transfer (LET) radiation [5,7]. LET is defined as the amount of energy that an ionizing particle transfers to the material it passes through, measured per unit distance. Under normal physiological conditions, Nrf2 undergoes regulation through its association with Kelch-like ECH-associated protein 1 (KEAP1), which leads to proteasomal degradation of Nrf2. However, under oxidative stress, Nrf2 dissociates from KEAP1, translocates to the nucleus and functions as an upstream transcription factor for various genes involved in antioxidant responses and ROS detoxification systems [8]. This mechanism serves as a protective shield for cells against ROS-induced damage [9] and facilitates DNA repair and survival [10]. Nrf2 effectively controls the expression of several genes essential for maintaining redox homeostasis, such as glutathione (GSH) and superoxide dismutase 1 (SOD1) [11,12]. The expression levels of several genes downstream of Nrf2 have been shown to be modified by irradiation [13]. Notably, it has been shown by us and other research groups that Nrf2 signaling plays an important role in resistance to radiation [11,14]; therefore, targeting Nrf2 signaling is potentially a way to overcome radioresistance in some tumors with poor prognoses, such as glioblastoma and pancreatic cancers [7,15].

New modalities in radiotherapy, such as particle therapy, are being proposed for the treatment of radioresistant tumors. In contrast to X-ray (low linear energy transfer, low LET) radiation, particle radiation (high linear energy transfer, high LET) releases most of its energy in a well-defined Bragg peak. This enables the administration of a high dose to the tumor and a low dose to the healthy tissues, which are localized in front as well as behind the Bragg peak [16], thus minimizing adverse effects on normal healthy tissues. Therefore, particle radiotherapy is a preferred treatment option for tumors located near critical organs, especially for pediatric patients with long survival times [17,18,19,20]. When compared to X-rays, particle radiotherapy demonstrates greater efficacy in eliminating targeted cells by causing a large amount of toxic multiple DNA damage sites [21], such as clustered DNA double-strand breaks (DSBs) [22]. Additionally, particle irradiation induces clustered oxidative DNA lesions that may lead to chronic oxidative stress [23], resulting in severe late effects on healthy tissues.

Most tissues contain a pool of stem cells that respond to trauma and damage. Exposure of healthy tissue to ionizing radiation includes exposure of the stem cells. Stem cells represent a crucial cellular component within normal tissue, indispensable for tissue maintenance, repair and regeneration. As part of the tissue repair process, the surviving stem cells are triggered and start to mobilize. Stem cells, which are naturally clonogenic, proliferate and replace the damaged or lost cells, thus repairing the lesion. Therefore, loss of stem cells by irradiation results in a reduced capacity to replace the functional cells. If an adequate number of stem cells survive in the irradiated region or its vicinity, a sufficient number of functional cells will be produced and complete healing will be observed.

However, high-dose radiation exposure will cause a substantial loss of stem cells. If the surviving stem cells are insufficient to produce a sufficient number of functional cells to replace the lost ones, an imbalance between cell loss and regeneration will occur, leading to permanent damage. The exceptional biological characteristics of stem cells, notably their ability to self-renew and differentiate into diverse specialized cell types, render them highly susceptible to the deleterious effects of radiation exposure. The adverse impact of radiation on stem cells can disrupt normal tissue regeneration processes, impair organ function and contribute to long-term health consequences. Understanding how radiation affects the behavior and fate of stem cells is crucial for protecting and enhancing tissue regeneration in individuals exposed to radiation. By exploring this area, scientists can devise strategies to support and strengthen stem cells, leading to improved recovery of damaged tissues in affected individuals.

Although low levels of ROS production are required for stem cells to undergo self-renewing, proliferation and proper differentiation [24,25,26,27], increased production of ROS is detrimental to stem cells and has been implicated in the pathogenesis of several pathological conditions. The pathological effects of ROS on stem cells are dose dependent. A moderate increase in ROS production can impair stem cell self-renewal by promoting proliferation and differentiation, resulting in the premature exhaustion of stem cells [28,29,30,31,32].

The aim of this study was to investigate the impact of Nrf2 inhibition combined with ionizing radiation on stem cell survival and the ability of surviving radiation-exposed stem cells to differentiate into osteogenic and adipogenic lineages.

To address this aim, we used adipose-derived stem cells (ADSCs) as experimental models. ADSCs were used as models because they have gained significant attention in regenerative medicine and tissue engineering due to their abundance, ease of isolation and remarkable regenerative potential. ADSCs possess the ability to differentiate into various cell lineages, including adipocytes, chondrocytes, osteocytes and myocytes. ADSCs exhibit enhanced survival rates and maintain their proliferative capacity even after exposure to moderate doses of radiation [33,34]. In this study, ADSCs were exposed to X-rays, protons or carbon ions. Stem cell survival and differentiation potential of the surviving stem cells were used as endpoints. The role of Nrf2 in the survival, proliferative capacity and stemness of ADSCs after exposure to X-rays, protons or carbon ions was evaluated by treating the cells with an Nrf2 inhibitor.

## 2. Materials and Methods

### 2.1. Cell Culture

ADSCs were purchased from Sigma-Aldrich (Saint-Quentin-Fallavier, France). 250,000 cells were cultured in 10 mL of Dulbecco’s modified Eagle medium (DMEM) (Dutcher, Bernolsheim, France), supplemented with 10% of fetal bovine serum (Dutcher, Bernolsheim, France) 1% PenStrept (10.000 U penicillin and 10 mg streptomycin/mL, Merck Life Science, Solna, Sweden) and 20 ng/mL of basic fibroblast growth factor (FGF) (Peprotech, ThermoScientific, Waltham, MA, USA) in T75 flasks. The medium was changed every 3.5 days. Every 7 days (at 80% confluence), the cells were washed three times with 5 mL of phosphate-buffered saline (PBS) without calcium and magnesium (Merck Life Science, Solna, Sweden), then incubated for 5 min at 37 °C with 1.5 mL of accutase solution (632 U/mL activity, Merck Life Science, Solna, Sweden) to detach the cells. After detachment, 250,000 cells were transferred to new T75 flasks with 10 mL of medium and incubated for another 7 days. This procedure was repeated three times, every 7 days. The cells were then aliquoted into cryotubes at 500,000 cells per tube and frozen at −150 °C in complete medium with 10% dimethyl sulfoxide (DMSO, Merck Life Science, Solna, Sweden). An aliquot was thawed and used to initiate a new experiment. The percentage of cells expressing lysosomal beta-galactosidase (senescence-associated beta-galactosidase, SA-beta-gal) was analyzed to determine the levels of senescent cells in the culture following the same protocol we previously published [35].

### 2.2. Nrf2 Inhibitor

The Nrf2 inhibitor ML385 was purchased from Selleckchem, Houston, TX, USA. It was then dissolved in DMSO (Merck Life Science, Solna, Sweden) using an ultrasonic bath for 10 min at 50 °C, achieving a final concentration of 10 mmol/L. The stock solution was then filtered, aliquoted and stored at −80 °C until use.

The Western blot (WB) technique was employed to study the efficacy of the Nfr2 inhibitor as well as the expression of alkaline phosphatase as an endpoint for osteogenesis. The expressions of heme oxygenase-1 (HO-1) and NAD(P)H quinone dehydrogenase (NQO1) proteins downstream of Nrf2 transcriptional activities were used as endpoints for the efficacy of the Nrf2 inhibitor.

### 2.3. Cell Preparation

ADSCs at passage 3 were thawed and cultured up to passage 6 to obtain the required number of cells. The cells were then equally divided into control and test groups. The control group was treated only with the vehicle, DMSO, at a concentration of 0.03%. The test group was treated with 6 μmol/L of the Nrf2 inhibitor. The concentration (6 μmol/L) and treatment duration (76 h before irradiation and 5 days after) were selected based on a protocol we established for treating glioblastoma stem cells in vitro (manuscript under review). Both control and test group cells were incubated at 37 °C with 5% CO_2_ for 76 h and then detached from the flasks using accutase as described earlier. The cells were counted, and 75,000 cells were transferred into an appropriate number of 2 mL Eppendorf tubes.

**Figure 1:**
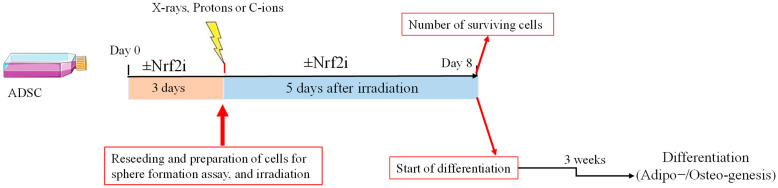
Experimental setup to study the effect of Nrf2i and radiation quality on the survival of ADSCs.

Prior to irradiation (Figure 1), the cells were washed 3 times with PBS without calcium and magnesium (Merck Life Science, Solna, Sweden) and dissociated from the bottom of culture flasks with accutase (Merck Life Science, Solna, Sweden) and, solved in 2 mL complete medium. 100 µL of cell suspension were mixed with 100 uL of ready-to-use trypan blue solution (Thermo Scientific, Waltham, MA, USA) and loaded onto a Malassez counting chamber (Glaswarenfabrik Karl Hecht, Sondheim, Germany). The cells were scored and counted using light microscopy. Approximately 75,000 cells from control or test groups were suspended in the corresponding 1.5 mL medium with or without inhibitor in 2 mL tubes, kept on ice and exposed to 0, 0.5, 1, 2 and 4 Gy of X-rays, protons or carbon ions. Two tubes per dose were used for each experiment and each experiment was repeated 3 times. Overall, for the three radiation qualities, 180 tubes were used: 90 tubes for the control group without inhibitor and 90 tubes for the test group with inhibitor. Following exposure, the 1.5 mL cell suspension was equally divided into 3 wells of a 6-well plate and then 3.5 mL complete medium with inhibitor (test group) or without inhibitor (control group) was added to each corresponding well of the 6-well plates. The plates were then incubated at 37 °C with 5% CO_2_ for 5 days after irradiation, washed with PBS (without calcium and magnesium) (Merck Life Science, Solna, Sweden), and disassociated from the culture flasks with accutase. The numbers of viable cells were counted by trypan blue dye to establish the dose–response relationship of survival. Comparing the level of survival in a test group relative to a control gives us information about the cytotoxicity of the treatment. A total of 90 plates were used, 45 for the control and 45 for the test groups.

For the Western blot analysis, 500,000 cells were treated for 5 days with inhibitor or vehicle in T75 flasks and then washed 3 times with PBS without calcium and magnesium (Sigma Aldrich) and disassociated from the bottom of culture flasks with accutase (Sigma Aldrich). The cells were counted using a Malassez counting chamber, as described earlier. Almost 1 × 10^6^ cells were taken and lysed for Western blot (WB) analysis.

### 2.4. ADSC Differentiation

Approximately 20,000 and 200 surviving cells five days after irradiation were seeded in triplicate in 6-well and 96-well plates with complete culture medium without Nrf2i for osteogenesis and adipogenesis, respectively, to test the ability of the cells to differentiate. 24 h later, the complete culture medium was replaced with osteogenic or adipogenic differentiation media (Figure 1). The osteogenic kit (Thermo Fisher Scientific, Grand Island, NY, USA) contained αMEM supplemented with 0.1% antibiotics (10.000 U penicillin and 10 mg streptomycin/mL, Merck Life Science, Solna, Sweden), 10% FBS, 100 nmol/L dexamethasone, 50 μg/mL ascorbic acid-2 phosphate and 10 μmol/L β-glyceraldehyde, all purchased from Fisher Scientific. The adipogenic kit (Thermo Fisher Scientific, Grand Island, NY, USA) contained αMEM with 0.1% antibiotics (10.000 U penicillin and 10 mg streptomycin/mL, Merck Life Science, Solna, Sweden), 10% FBS, 100 nmol/L dexamethasone and 0.5 mmol/L isobutyl-methylxanthine. For osteogenesis, the medium was replaced twice per week. After 21 days, osteocytes were fixed using 4% paraformaldehyde (PFA) and the levels of deposited calcium were stained with 2% alizarin red solution (Merck Life Science, Solna, Sweden). Alizarin red solution was then extracted using 2 mL of 10% acetic acid. The extracted alizarin red was then added to 4 wells of a 96-well plate. The intensity of the red color was then measured by an automatic 96-well plate reader at 405 nm. The signals from non-irradiated cells were set as 1 and the signals from irradiated samples were calculated relative to the signals from non-irradiated cells. The levels of osteocytes were also checked by analyzing alkaline phosphates using Western blot analysis.

Adipocytes, on the other hand, were fixed with 4% PFA after 21 days and then 100 µL of Oil Red solution (Merck Life Science, Solna, Sweden) was added to each well of the 96-well plate to stain lipid droplets. The cells were washed 3 times with PBS and then stained for 45 s with a hematoxylin and eosin staining kit (Abcam, Cambridge, UK). Examples of stained cells are presented in Figure S1. At least 200 cells per well were scored by inverted light microscopy at 10× magnification following the previously published article [36]. The numbers of cells containing lipid droplets (mature adipocytes) relative to the number of cells without lipid droplets were then calculated and expressed as percentages of mature adipocytes in the cell population. Examples of mature differentiated adipocytes are presented in Figure S1.

### 2.5. Irradiations

For X-ray irradiation, CellRad^®^ Faxitron irradiator, available at Cyceron Platform, Caen, France, was used. The voltage was set to 125 kV, intensity to 4.7 mA, and an external 0.3 mm copper filter was used. The dose rate was 2 Gy/min.

Irradiation with carbon ions was carried out as described in our earlier study [7] using a carbon ion beam at the National Large Heavy Ion accelerator (GANIL, Caen, France) in the D1 experimental area using the IRABAT horizontal beamline managed by the interdisciplinary research CIMAP–CIRIL platform. Samples were irradiated by a carbon ion beam in 2 mL tubes with an initial energy of 95 MeV/nucleon. The native beam had a LET of 28 keV/μm and, by using a polymethyl methacrylate degrader, a LET of 33 keV/μm was obtained at the place where the cells were exposed. The dose rate was approximately 2 Gy/min.

For proton irradiation, a pencil beam scanning technique (PBS) was used. The cells were exposed to protons in the plateau of the spread-out Bragg peak (SOBP) with energy ranges between 110 MeV to 129 MeV, creating a 2 cm SOBP at 9–11 cm depth. Irradiation was performed using the ProteusOne IBA cyclotron in the CYCLHAD proton therapy center in Caen, France. The beam direction was horizontal. A dose rate of 2 Gy/min was used. Irradiation was performed using a specific holder for 2 mL Eppendorf tubes positioned at 10 cm depth in a box made of 5 × 15 × 15 cm tissue-equivalent plexiglass. The dosimetry and dose calculations were performed by medical physicists at the proton therapy center using the proton RayStation treatment planning system (RaySearch, Uppsala, Sweden).

For calculation of LD50 (the dose of radiation that is expected to cause death in 50 percent of the exposed cells), mathematical functions based on a linear-quadratic model for each dose-response relationship were established [37]. The RBE was calculated as a ratio of the LD50 of X-rays as the reference radiation quality to the LD50 of protons or carbon ions.

### 2.6. Preparation of Cells for Western Blotting (WB)

48 h after irradiation, the cells were lysed in standard RIPA (radioimmunoprecipitation assay) buffer supplemented with a protease inhibitor cocktail (ThermoScientific, Waltham, MA, USA). We applied the present protocol for Western blotting in our previous publication [7]. Briefly, proteins were quantified using Pierce protein assay reagent (ThermoScientific, Waltham, MA, USA) according to the manufacturer’s instructions, and 10 μg of total protein and 2 μL of molecular weight markers (Thermo Fisher Scientific, Grand Island, NY, USA) were used for electrophoresis in NuPAGE 4–12% Bis–Tris pre-casted gel (ThermoScientific, Waltham, MA, USA) and migrated for 2 h at 100 V. Following electrophoresis, the proteins were transferred onto a PVDF (polyvinylidene difluoride) membrane (Thermo Fisher Scientific, Grand Island, NY, USA) using the XCell SureLock™ Mini Cell system (Thermo Fisher Scientific, Grand Island, NY, USA) for 2 h at 30 V on ice. Samples were incubated in blocking buffer (Thermo Fisher Scientific, Grand Island, NY, USA), washed and then incubated with the following primary antibodies: anti-CD73 from rabbit at 1:800 (Merck Life Science, Solna, Sweden); anti-HO-1 from mouse at 1:1000 (Santa Cruz Biotechnology, CA, USA), anti-NQO1 from rabbit at 1:1000 (Merck Life Science, Solna, Sweden), anti-alkaline phosphatase from rabbit (Abcam, Cambridge, UK) and anti-GAPDH from mouse at 1:10000 (Merck Life Science, Solna, Sweden), as well as anti-GAPDH from rabbit (when appropriate) at 1:1000 (Merck Life Science, Solna, Sweden). Primary antibodies were diluted in the blocking buffer, and the incubation took place overnight at 4 °C. Secondary antibodies were anti-mouse HRP (Horseradish peroxidase) conjugated and anti-rabbit HRP conjugated (ThermoScientific, Waltham, MA, USA). Immobilion^®^ Crescendo Western HRP substrate (Thermo Fisher Scientific, Grand Island, NY, USA) was added to detect the signals of the HRP-conjugated secondary antibody. Thereafter, the membranes were scanned in the Azure imaging system. Quantification analysis of Western blot bands was performed using Image Studio ver. 5.2 software.

### 2.7. Statistics

Data are presented as mean ± standard deviation, *n* = 3. Statistical analyses were performed by Student’s *T*-test and two-way ANOVA with Tukey’s multiple comparison test to compare different sets of data using Graph Pad prism 7 software. A *p* < 0.05 was deemed significant and flagged with 1 star (*). If *p* < 0.01, it was flagged with 2 stars (**) and if *p* < 0.001, it was flagged with 3 stars (***).

## 3. Results

### 3.1. Efficiency of the Nrf2 Inhibitor

**Figure 2:**
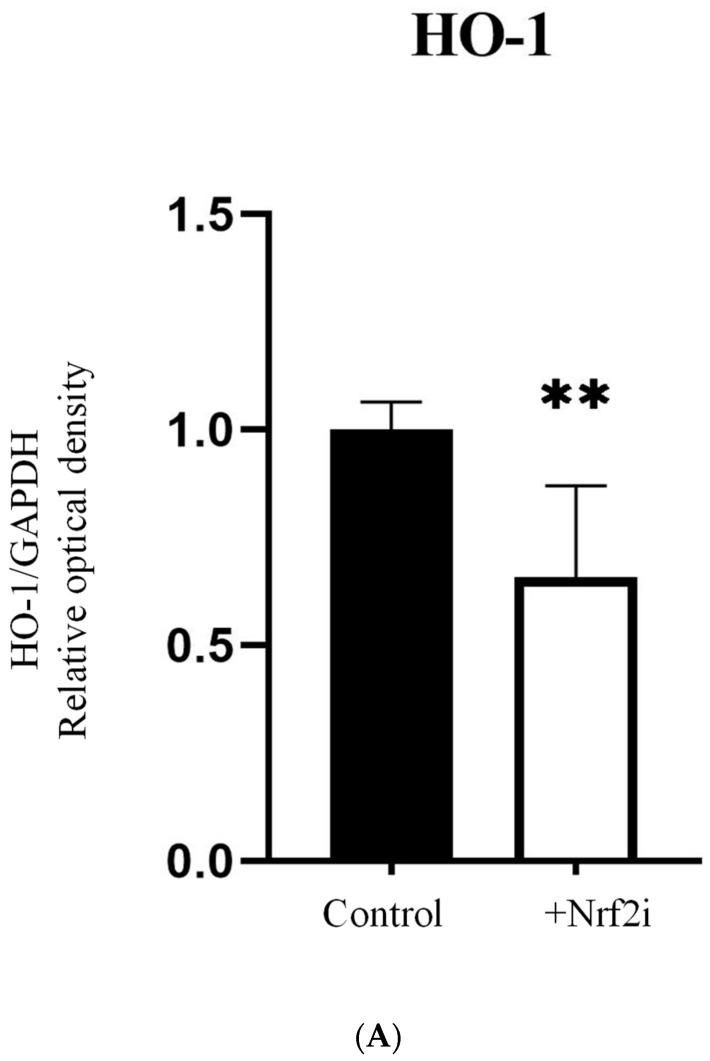

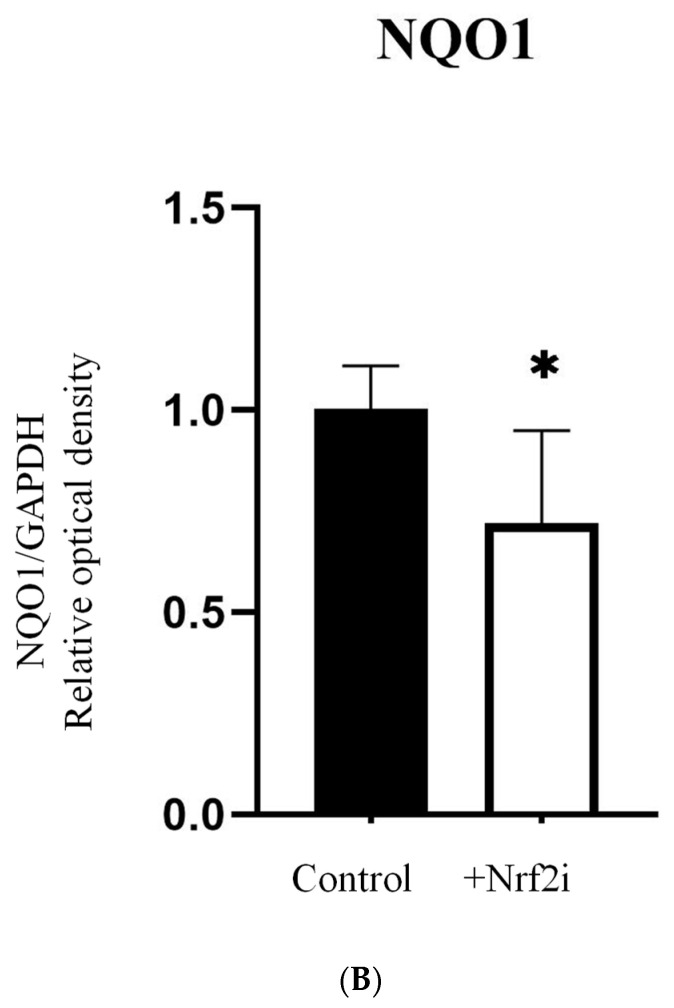
Quantitative analysis of HO-1 (**A**) and NQO1 (**B**) proteins by Western blot in ADSC, 5 days after treatment with Nrf2i. A paired *t*-test was performed. The values are presented as mean ± standard deviation, *n* = 6, *; *p* < 0.05, **; *p* < 0.001.

A significant decrease in the expression of proteins HO-1 and NQO1 was observed in the ADSCs treated with Nrf2 inhibitor (test group) as compared with non-treated cells (control group), indicating the functionality of the inhibitor (Figure 2A,B). The images of the Western blot membranes are shown in Supplementary Figure S3A,B.

### 3.2. Dose–Response Relationship of Survival after Exposure to Radiation Quality

**Figure 3:**
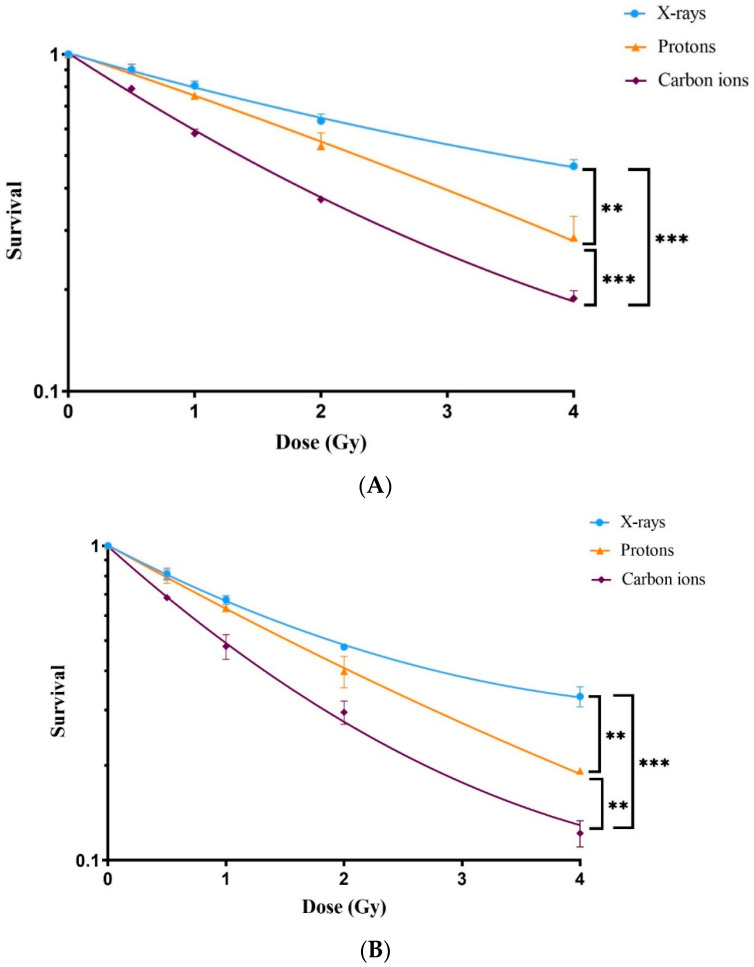
Dose–response relationships of survival of adipose-derived stem cells (ADSCs) in the absence (**A**) and presence (**B**) of Nrf2i (ML385) in response to different radiation qualities. The survivals were established by staining cells with trypan blue dye and counting viable cells. The values are presented by mean ± standard deviation, *n* = 3, **; *p* < 0.001, ***; *p* < 0.0001.

A statistically significant reduction in the survival of ADSCs was observed following exposure to all three radiation qualities. The greatest reduction was observed after exposure to carbon ions (Figure 3A), followed by protons and X-rays, respectively. The LD50 value for X-rays was 3.5 ± 0.08 Gy, protons 2.23 ± 0.27 Gy and carbon ions 1.61 ± 0.05 Gy. The calculated RBEs of protons and carbon ions were 1.6 ± 0.191 and 2.2 ± 0.12, respectively.

Treatment of ADSCs with the Nrf2 inhibitor (ML385) prior to irradiation led to a profound reduction in ADSC survival following exposure to X-rays, protons and carbon ions (Figure 3B). The presence of the Nrf2 inhibitor resulted in further reduction in LD50 values compared to those without the Nrf2 inhibitor. LD50 values decreased to 2.33 ± 0.13 Gy after X-rays, to 1.60 ± 0.06 Gy after protons and to 1.24 ± 0.03 Gy following carbon ion exposure. Analysis of the sensitizing ratio (LD50 Nrf2 inhibitor/LD50 control) of the cells revealed a significantly greater sensitivity to Nrf2 inhibition in response to X-rays (33%) compared to protons (28%) and carbon ions (23%), as shown in Table 1.

### 3.3. ADSC Differentiation after Radiation

**Figure 4:**
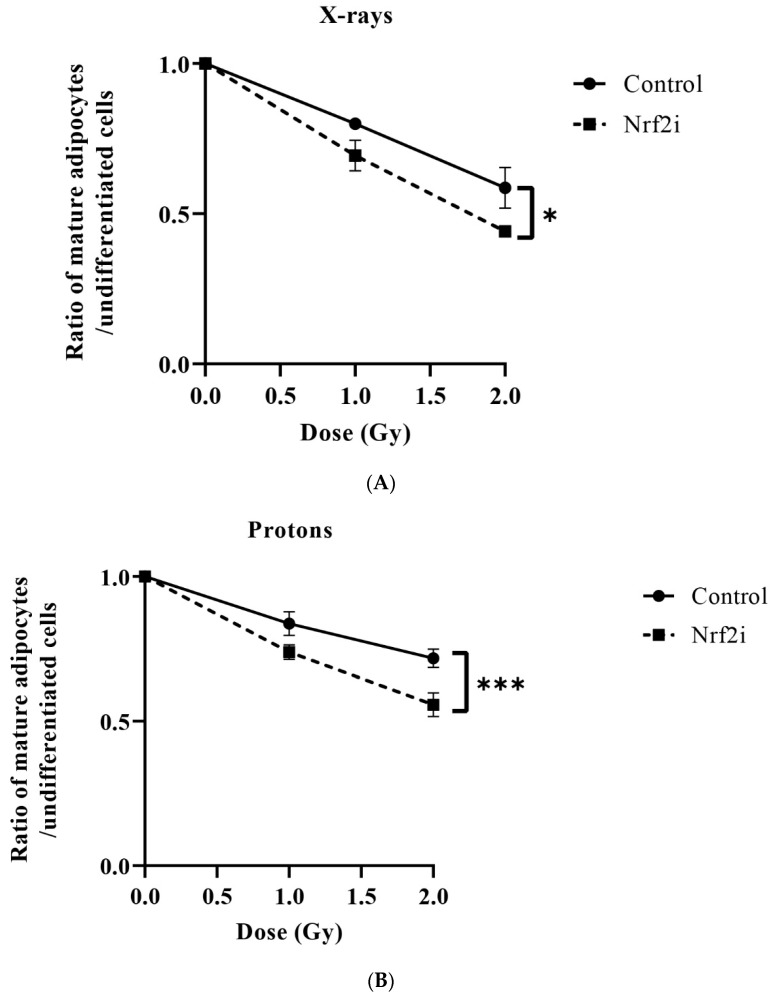

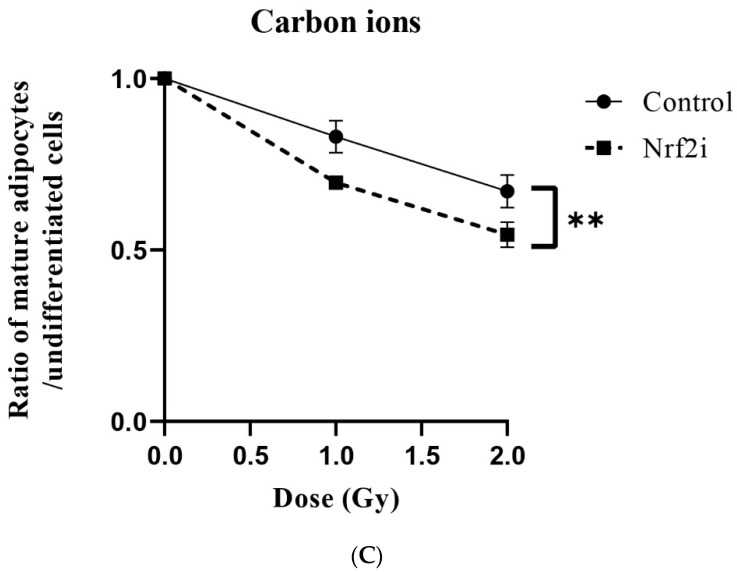
Ratio of mature adipocytes to undifferentiated ADSCs in the absence (●) and presence of Nrf2i (■) exposed to different radiation qualities: X-rays (**A**), protons (**B**) and carbon ions (**C**). The values are presented as mean ± standard deviation, *n* = 3, *; *p* < 0.05, **; *p* < 0.001, ***; *p* < 0.0001.

Adipogenesis decreased significantly when cells were exposed to X-ray radiation (Figure 4B,C and Table 2). When the Nrf2 inhibitor was added, a reduction in adipogenesis was evident in all three types of radiation qualities (Figure 4A–C). The DI50, which indicates the dose required to inhibit differentiation by 50%, was found to be highest for protons at 3.95 ± 0.08 Gy, followed by carbon ions at 3.61 ± 0.24 Gy and then X-rays at 2.69 ± 0.43 Gy. The results indicate that X-rays affect adipogenesis negatively at lower doses than those of carbon ions and protons. In the presence of Nrf2 inhibitor, the DI50 doses were decreased to 1.76 ± 0.08 Gy for X-rays, 2.4 ± 0.09 Gy for protons and 2.23 ± 0.13 Gy for carbon ions (Table 2). These results revealed radiosensitizing effects similar to those of Nrf2i, approximately 36%, for all radiation qualities (Table 2).

**Figure 5:**
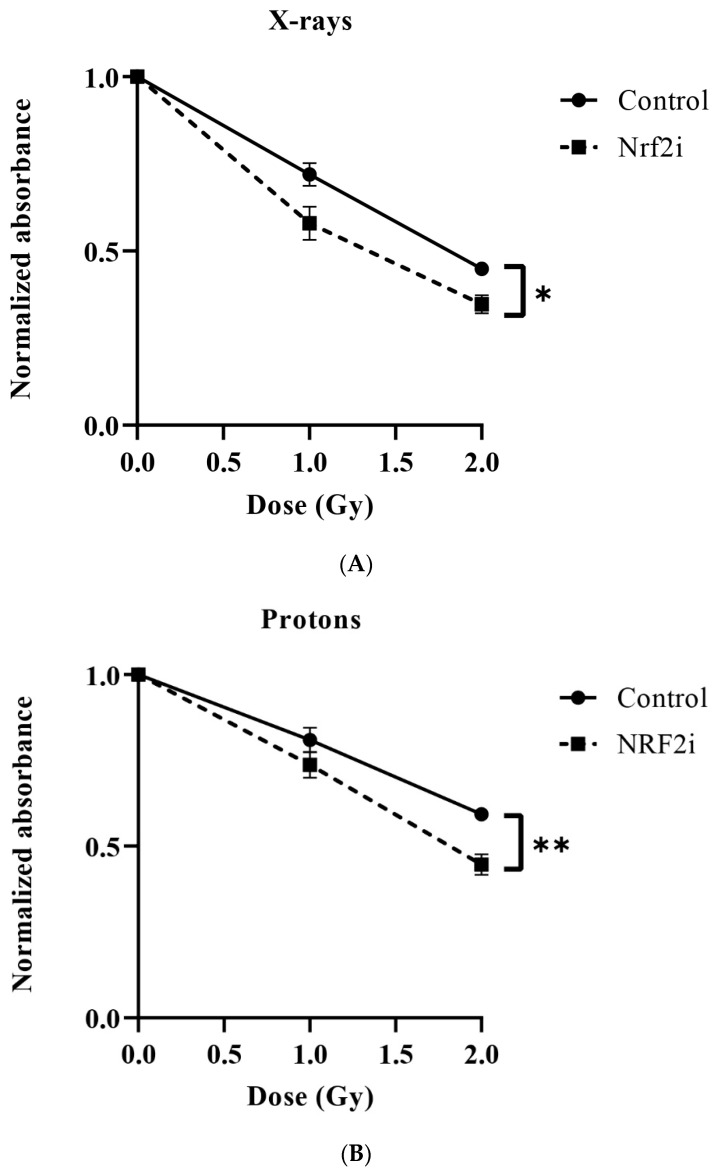

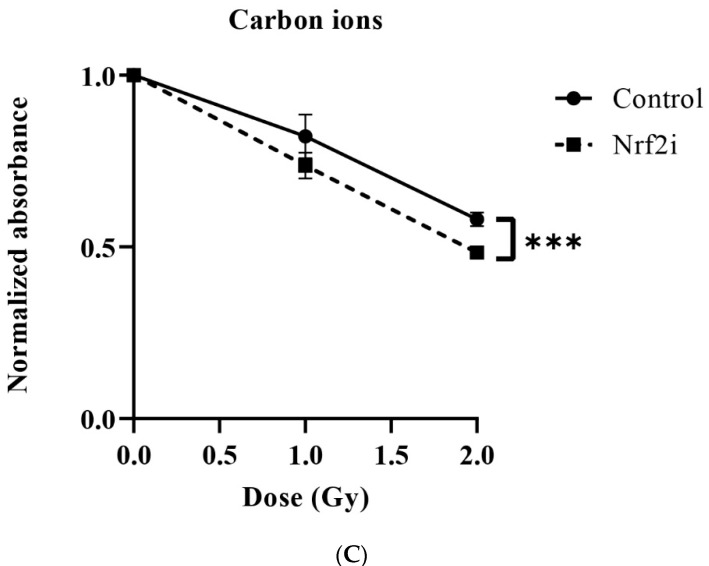
The effects of radiation quality and Nrf2i on osteogenesis. Normalized optical densities at 405 nm after alizarin red staining in the absence (●) and presence of Nrf2i (■) after different radiation qualities X-rays (**A**), protons (**B**) and carbon ions (**C**). The values are presented by mean ± standard deviation, *n* = 3, *; *p* < 0.05, **; *p* < 0.001, ***; *p* < 0.0001.

The results of osteogenesis are summarized in Figure 5. They indicate a significant decrease of osteogenesis after exposure to radiation and adding Nrf2i further decreased the osteogenesis, as determined by alizarin red staining (Figure 5A–C, and Table 3). The DI50 for osteogenesis was higher for proton radiation (2.76 ± 0.208 Gy) than for carbon ion radiation (2.69 ± 0.057 Gy) and X-ray radiation (1.79 ± 0.052 Gy). Treatments of the cells with Nrf2i and radiation resulted in further reduction of the osteogenesis as follows: DI50 for carbon ions 1.96 ± 0.061 Gy, DI50 of protons 1.91 ± 0.191 Gy and DI50 of X-rays 1.30 ± 0.107 Gy (Table 3). These results revealed radiosensitizing effects similar to those of Nrf2i, approximately 28–30%, in all radiation qualities (Table 3).

**Figure 6:**
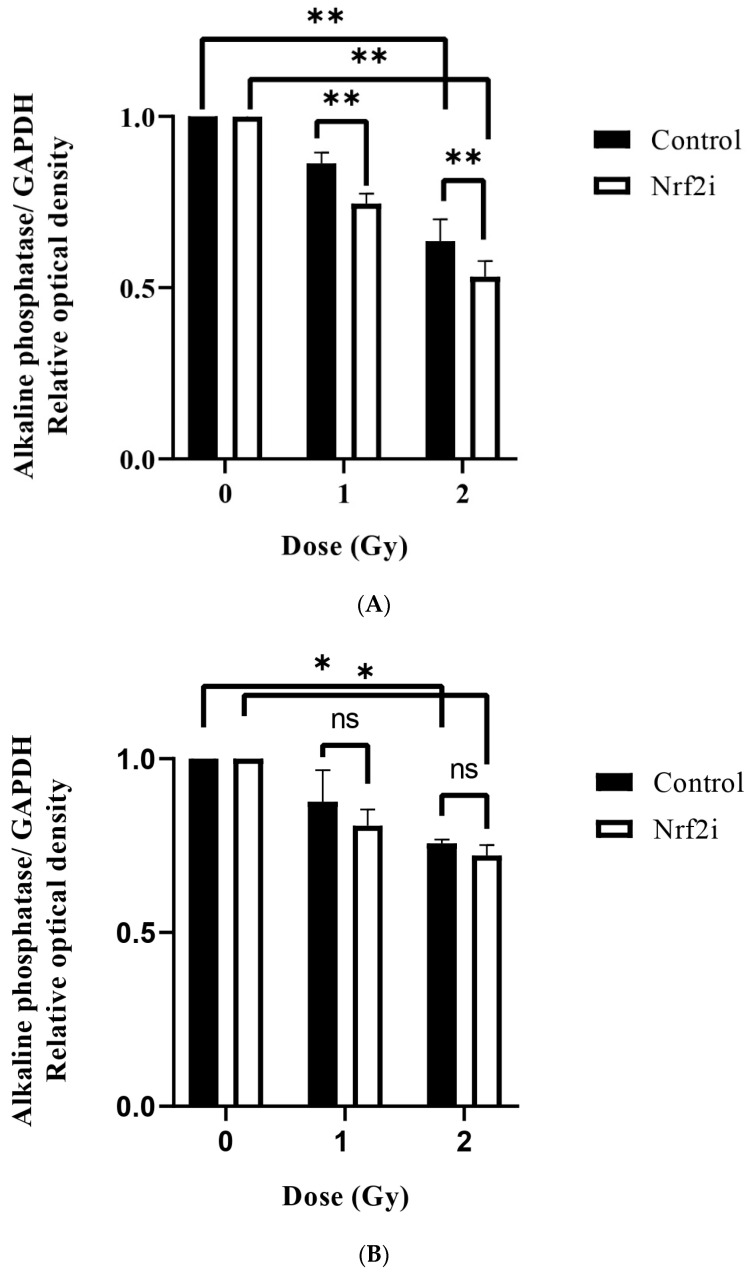

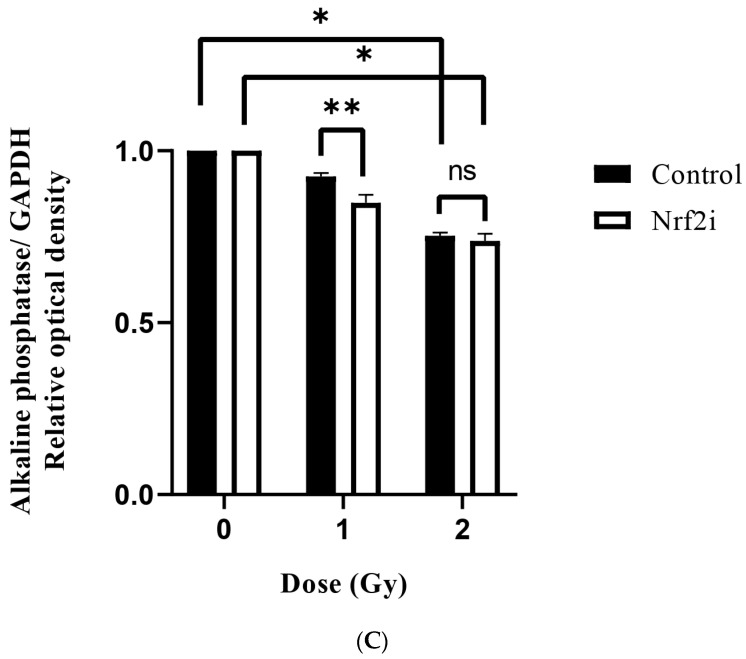
Alkaline phosphatase expression determined by Western blotting 21 days after osteogenesis of ADSCs in the presence (black bars) and the absence of Nrf2i (white bars) after exposure to the different radiation qualities of X-rays (**A**), protons (**B**) and carbon ions (**C**). The values are presented by mean ± standard deviation, *n* = 3, *; *p* < 0.05; **; *p* < 0.001 and ns: no significant change.

A significant decline in ALP levels following exposure to 1 Gy (without inhibitor) (0.86 ± 0.03, 0.87 ± 0.09, 0.92 ± 0.01) and 2 Gy (0.64 ± 0.06, 0.76 ± 0.01, 0.75 ± 0.01) of X-rays, protons and carbon ions, respectively, was observed (Figure 6A–C). Adding Nrf2i further decreased the ALP levels significantly by 1 Gy for X-rays (from 0.86 ± 0.03 to 0.74 ± 0.03) (Figure 6A), and carbon ions (from 0.92 ± 0.01 to 0.85 ± 0.02) (Figure 6C) and 2 Gy for X-rays only (Figure 6A), (from 0.64 ± 0.06 to 0.53 ± 0.05) indicating the involvement of Nrf2 in stemness and osteogenic differentiation, particularly for X-rays. An example of the Western blot image is shown in Supplementary Figure S3C.

These observations highlight potential differences in the osteogenic response of ADSCs to various radiation qualities and Nrf2i, emphasizing the urgency for further examination of specific molecular mechanisms involved in osteogenesis.

## 4. Discussion

The effects of ionizing radiation on bone marrow and neuronal stem cell survival and differentiation have already been reported in multiple studies [38,39,40], but less is known about how radiation affects ADSCs. Exposure to radiation can result in DNA damage and trigger programmed cell death (apoptosis) or can sublethally damage stem cells, ultimately decreasing their capacity for regeneration and therapeutic effectiveness [41]. This might be important in the development of radiotherapy-induced late severe side effects, particularly for cancer patients with long-term survival.

The present study investigates the impact of different radiation qualities on the survival and stemness of ADSCs. These cells are important for the repair of radiation-induced healthy tissue damage. The results presented in Figure 3A and summarized in Table 1 show that the LD50s for X-rays, protons and carbon ions are 3.5 Gy, 2.2 Gy and 1.6 Gy, respectively. This indicates a higher cytotoxicity for carbon ion radiation compared to protons and X-rays. The enhanced cytotoxicity of carbon ions can be attributed to the induction of a greater amount of complex DNA damage than with low-LET X-rays or protons. Complex DNA damage is defined as two or more lesions within one or two helical turns of the DNA and plays a significant role in radiation cytotoxicity due to the difficult nature of its repair by the cells [42,43]. This supports the findings of Perez et al. [44], who reported similar findings for carbon ions, and the findings of Chaudhary et al. (2014) [45] and others who reported similar findings for protons [46,47].

In a previous study, it was shown that ADSCs can form colonies, which makes it possible to perform a colony-forming assay with ADSCs [48]. The colony-forming assay is considered the gold standard for survival. In our study, we used a trypan blue exclusion assay rather than a colony-forming assay because the ADSCs used were unable to form colonies. One important explanation might be that in the study by Schröder et al. [48], a colony-forming assay was performed immediately after the isolation of ADSCs when the cells were in low passage. ADSCs are primary and enter the senescence stage after a certain number of cell divisions, thus limiting their proliferation capacity [49]. In our study, the ADSCs were at passage 4, and although they were unable to form colonies, they were not fully in the senescence stage (7% of the cell population, see Supplementary Table S1) and could still proliferate normally. Interestingly, Nrf2i did not significantly change the percentage of senescent cells. Further, counting the number of viable ADSCs by different methods in culture to investigate cytotoxicity of a certain toxin has been used in several other publications [50,51]

The calculated RBE for carbon ions was 2.2 and for protons 1.58, as shown in Table 1. The obtained RBE of carbon ions is within the range previously published [52]. In the literature, a fixed RBE of 1.1 has been suggested for protons [53]. In our study, a higher RBE for protons was obtained, which could be due to the proton delivery technique. We irradiated our cells using multienergetic clinical proton beams by pencil beam scanning techniques (PBS) in the SOBP area. Perhaps PBS gives an increased RBE compared with the passive scattered beam by collimator technique that has previously been used. This has been reported by Leduc A et al. [54]. Another explanation could be that our ADSCs were irradiated in the SOBP, where protons might have a higher RBE than 1.1. Most studies previously done for the calculation of proton RBE have been carried out with cells located in the entrance of the beam, where an RBE of 1.1 has been observed. A higher RBE of protons may also be related to the different cell and survival assays used in the present investigation. In the present work, cell numbers 5 days after irradiation were determined, while the gold standard for survival in radiation biology is the colony-forming ability test. Also, the cell type (e.g., stem cell vs. tumor cell) might play a role. However, not all the primary cells can form colonies and an alternative method has to be employed [55]. We have established a protocol based on agarose overlay to stimulate primary human fibroblasts forming colonies [55], but this did not work for ADSCs. Protons and carbon are believed to rely less than X-rays on the indirect effect of radiation involving ROS. Therefore, it would be more meaningful to compare the effects demonstrated in the present study based on isosurvival levels for the three radiation qualities.

Targeting Nrf2 signaling has been suggested by us and by other research groups to increase the sensitivity of radioresistant tumor cells to radiotherapy [7,56]. Generally, when using radiosensitizing drugs to increase the effect of radiotherapy, the drugs may also affect, perhaps increase, the normal cell response to the treatment [57,58]. Therefore, it is essential to investigate their effects on normal cells. In the present study, ADSCs were used as a model to study the effects of radiation and Nrf2i on primary stem cells. An Nrf2 inhibitor was used to partially inhibit the Nrf2 pathway in the ADSCs and investigate its effects on cell survival and stemness.

Under normal physiological conditions, Nrf2 activation protects cells from the harmful effects of ROS by upregulating antioxidant enzymes [11]. However, when Nrf2 is inhibited, cells may have a reduced antioxidant capacity to cope with high levels of ROS caused by the radiation and thus be more vulnerable to ROS-induced DNA damage. Recently, it was shown that lowering Nrf2 in cells and mice can increase their sensitivity to X-rays due to increased levels of ROS, both at steady-state levels and in response to radiation [59]. These findings indicate that the Nrf2 pathway plays a role in protecting the cells from endogenously produced ROS, as well as from exogenously produced ROS, e.g., by radiation [59]. The production of ROS induced by ionizing radiation during radiotherapy has the potential to activate Nrf2, which in turn could reduce the killing effects of radiation. It has been demonstrated that the inhibition of Nrf2 activity enhances the sensitivity to radiation of radioresistant triple-negative breast cancer (TNBC) stem cells (CSCs) and glioblastoma stem cells [7,60].

Today, particle radiotherapy, particularly proton therapy, is recommended for the treatment of pediatric cancer patients. Pediatric cancer patients have good outcomes from the treatment with long survival times. One important explanation for the recommendation is that the use of particle therapy delivers a high dose to the tumor and a low dose to the normal tissues in the vicinity of the tumor [61,62]. As a result, a reduced risk of radiotherapy-induced late effects, such as cerebrovascular and cardiovascular effects, in long-time cancer survivors is expected [61]. However, particle radiation induces complex DNA damage, not only in the tumor tissues but also in the normal tissues through which the radiation passes to reach the tumor cells; however, the dose to the normal cells, including stem cells, is low due to the Bragg peak. Extensive research has been performed to investigate the effects of X-rays on stem cells [33], but knowledge about the effects of particle radiation on ADSCs, particularly the differentiation ability of the surviving cells, is limited. A study by Kurpinsky et al. demonstrated that transcriptomic profiles (cell cycle regulation, DNA replication and cell proliferation) differed between cells exposed to high and low LET radiations, suggesting radiation-induced modifications of the proliferation and differentiation (osteogenesis) of the exposed mesenchymal stem cells [63].

Due to their metabolic activity, malignant cells exhibit higher basal levels of ROS than normal tissue cells. Consequently, they are more reliant on the continuous activation of Nrf2 signaling, resulting in elevated levels of antioxidants. It has been reported [64,65] that increased ROS levels suppress cell growth and increase apoptosis in cancer cells, which renders cancer cells more susceptible to high levels of ROS. The cytotoxicity of many chemotherapy compounds is due to increased ROS production [66,67]. The results in Table 1 show that the inhibition of Nrf2 signaling increased the sensitivity of the ADSC cells to X-rays by 33%, protons by 28% and carbon ions by 23%. The differences in the sensitizing effects of Nrf2i might be due to the different distributions and levels of ROS induced by different radiation qualities. It was shown that the specific distribution of ROS after high LET carbon ion irradiation can induce complex DNA damage in ion tracks, may preserve some intracellular structures outside the ion tracks and does not allow the achievement of the threshold of ROS that is necessary to activate the signaling pathways involved, for example, in migration [68] and perhaps in differentiation processes. The data suggest that, for survival, cells irradiated with X-rays are more dependent on the Nrf2 pathway than those irradiated with protons or carbon ions.

The results presented in Figure 4 and Table 2 show that X-rays can have a greater effect on ADSC adipognesis (DI50 2.7 ± 0.43 Gy) than proton (DI50 3.91 ± 0.08 Gy) and carbon ions (3.61 ± 0.23) radiation. For osteogenesis (Figure 5 and Figure 6 and Table 3), the observed DI50s were 1.79 ± 0.05 Gy for X-rays, 2.76 ± 0.21 Gy for protons and 2.69 ± 0.06 Gy for carbon ions. This could be due to differences in the distributions of ROS caused by low LET and high LET radiation. The levels and cellular distribution of ROS are essential for stem cell differentiation processes [69].

It has been shown that mesenchymal stem cells need certain levels of ROS for differentiation to adipogenic lineage [70,71]. Certain levels of ROS due to Nrf2 inhibition and irradiation may stimulate adipogenesis by activating peroxisome-proliferator-activated receptor gamma (PPARδ), a nuclear receptor that is involved in lipid metabolism and adipogenesis [72]. Our results, presented in Figure 4 and Figure 5, show that X-rays (without Nrf2i) reduce adipogenesis and osteogenesis more than protons and carbon ions. Pretreatment with Nrf2i further decreases adipogenesis and osteogenesis for all radiation qualities by almost 36% and 29%, respectively, indicating that the differentiation ability of surviving-irradiated-ADSCs is not affected by radiation quality.

Studies have shown that Nrf2 plays a crucial role in enhancing the differentiation capacity of preadipocytes. It was also reported that when exposed to ROS, the expression and activity of Nrf2 increased, further contributing to the accumulation of lipids in adipocytes. However, when Nrf2 was absent, the lipid accumulation was relieved [73]. This could be explained by an increased ROS level that facilitates the recruitment of Nrf2 to the SREBP-1 promoter, leading to the transcription of target genes and the subsequent promotion of lipogenesis. These results corroborate our findings and present a new perspective by revealing that Nrf2, as a crucial signaling factor, establishes a connection between oxidative stress and the initiation of fat accumulation in adipocytes.

In recent years, scientists have shown that the regulation of ROS levels involved in stem cell self-renewal [74] and differentiation [75] is particularly accomplished through Nrf2 signaling [76]. High levels of ROS inhibit signaling pathways, such as Wnt/catenin and NELL-1, thereby inhibiting osteogenesis [77]. X-ray radiation can negatively impact the ability of ADSCs to undergo osteogenesis, potentially through the involvement of the Nrf2 pathway. Inhibiting Nrf2 can adversely affect irradiated ADSCs. Conversely, increasing Nrf2 levels in nuclear extracts has been shown to efficiently differentiate human periodontal ligament cells toward the osteogenic lineage [78]. Additionally, a study of Nrf2-knockout mice has demonstrated a significant deficit in postnatal bone acquisition [79]. Therefore, it can be concluded that Nrf2 is a crucial factor in the maintenance of ADSCs and their differentiation into the osteogenic lineage. When Nrf2 signaling is compromised, ADSCs’ self-renewal and differentiation into the osteogenic lineage are affected negatively. While radiation can enhance this effect, the type of radiation has no or minimal impact on this process.

It is essential to emphasize the significant role of Nrf2 in preserving stemness [80]. Our findings show that the protein expression of CD73 (a positive marker for ADSCs) tends to be constant after different X-ray and carbon ion doses 48 h after irradiation. CD73 expression tends to decrease slowly upon Nrf2 inhibition after both X-ray and carbon ion radiation, indicating that the presence of Nrf2 preserves ADSC stemness (Supplementary Figure S2).

## 5. Conclusions

Considering that the tumor cells are more sensitive than normal tissue cells to high levels of ROS and have a limited capacity to combat oxidative stress [81], it can be speculated that perhaps inhibiting (to some extent) the Nrf2 pathway can bring about a therapeutic gain for cancer cells over normal tissues. On the other hand, the stem cells studied in this report show a LET-dependent response (Table 1) to Nrf2 inhibition. This implies that Nrf2 activity is more pronounced when the cells are irradiated with X-rays than with protons and carbon ions. Interestingly, our results on the differentiation abilities of surviving stem cells (Figure 4, Figure 5 and Figure 6 and Table 2 and Table 3) indicate that the proportion of cells that survive carbon ions or proton irradiation, with or without Nrf2i, and can differentiate into adipocyte and osteocyte lineages, is significantly higher than that of surviving X-ray-irradiated cells. Therefore, we can conclude that using carbon ions or protons can affect the stemness of irradiated ADSCs at lower levels than those of X-rays, thus being more beneficial for long-time cancer survivors.

## Figures and Tables

**Table 1:** The effects of radiation quality and Nrf2 inhibitor on the survival of ADSCs. The data are expressed as mean ± standard deviation, *n* = 3, **; *p* < 0.001, ***; *p* < 0.0001. X: X-rays, P: protons, C: carbon ions, +Nrf2i: cells treated with Nrf2 inhibitor, −Nrf2i: cells not treated with Nrf2 inhibitor, LD50: dose that kills 50% of the cells, RBE: relative biological effectiveness.

Radiation Quality	LD50		*p* Value		RBE		Sensitizing Effect (%)
	−Nrf2i	+Nrf2i	−Nrf2i	+Nrf2i	−Nrf2i	+Nrf2i	LD50 (+Nrf2i)/LD50 (−Nrf2i)
X-rays	3.5 ± 0.08	2.33 ± 0.13	X-P < 0.003 **	X-P 0.003 **	1	1	33
Protons	2.23 ± 0.27	1.60 ± 0.27	P-C < 0.001 ***	P-C 0.003 **	1.57 ± 0.19	1.45 ± 0.1	28.25
Carbon ions	1.61 ± 0.05	1.24 ± 0.03	X-C < 0.001 ***	X-C < 0.001 ***	2.19 ± 0.12	1.88 ± 0.09	23

**Table 2:** The effect of radiation quality and Nrf2i on adipogenesis. DI50 (dose that inhibits differentiation of ADSC by 50%) and RBE of adipogenesis. The *p* values indicate the significance of the effects of Nrf2i at different radiation qualities. The values are presented by mean ± standard deviation, *n* = 3, **; *p* < 0.001, ***; *p* < 0.0001. +Nrf2i: cells treated with Nrf2 inhibitor, −Nrf2i: cells not treated with Nrf2 inhibitor, DI50: dose that inhibits differentiation of the ADSC by 50% and RBE: relative biological effectiveness.

Radiation Quality	DI50		*p* Value	RBE		Sensitizing Effect (%)
	−Nrf2i	+Nrf2i		−Nrf2i	+Nrf2i	DI50 (+Nrf2i)/DI50 (−Nrf2i)
X-rays	2.69 ± 0.43	1.76 ± 0.08	0.022 **	1	1	34.57
Protons	3.95 ± 0.08	2.42 ± 0.09	0.0018 ***	0.68 ± 0.08	0.72 ± 0.06	38.74
Carbon ions	3.61 ± 0.24	2.23 ± 0.13	0.019 **	0.74 ± 0.11	0.78 ± 0.04	38.2

**Table 3:** DI50 and RBE of osteogenesis of ADSC and their *p* values at different radiation qualities in the absence and presence of Nrf2i, expressed as mean ± standard deviation. The values are presented by mean ± standard deviation, *n* = 3, **; *p* < 0.001, ***; *p* < 0.0001. +Nrf2i: Nrf2 inhibitor treated group, −Nrf2i: group not treated with Nrf2 inhibitor, DI50: dose that inhibits differentiation of the ADSC by 50% and RBE: relative biological effectiveness.

Radiation Quality	DI50		*p* Value	RBE		Sensitizing Effect (%)
	−Nrf2i	+Nrf2i		−Nrf2i	+Nrf2i	DI50 (+Nrf2i)/DI50 (−Nrf2i)
X-rays	1.79 ± 0.052	1.30 ± 0.11	0.002 **	1	1	27.37
Protons	2.76 ± 0.208	1.91 ± 0.19	0.002 **	0.65 ± 0.08	0.68 ± 0.06	30.72
Carbon ions	2.69 ± 0.057	1.96 ± 0.06	0.0001 ***	0.66 ± 0.11	0.66 ± 0.04	27.13

## Data Availability

The data are available upon request.

## Supplementary Materials

The following supporting information can be downloaded at: https://www.mdpi.com/article/10.3390/antiox13091035/s1, Figure S1: Examples of differentiated adipocytes. Figure S2: Expression of CD73 protein. Figure S3: Examples of western blot images. Table S1: Percentage of S-beta galactosidase positive cells.
