# Supplementary material for: Role of Oxidative Stress Signaling, Nrf2, on Survival and Stemness of Human Adipose-Derived Stem Cells Exposed to X-rays, Protons and Carbon Ions
Source: Antioxidants (Basel). 2024 Aug 26;13(9):1035. doi: 10.3390/antiox13091035 (PMC11429097; doi:10.3390/antiox13091035)
Supplement: Supplementary file 1 [file antioxidants-13-01035-s001.zip › antioxidants-3099896-supplementary.pptx]

## Slide 1
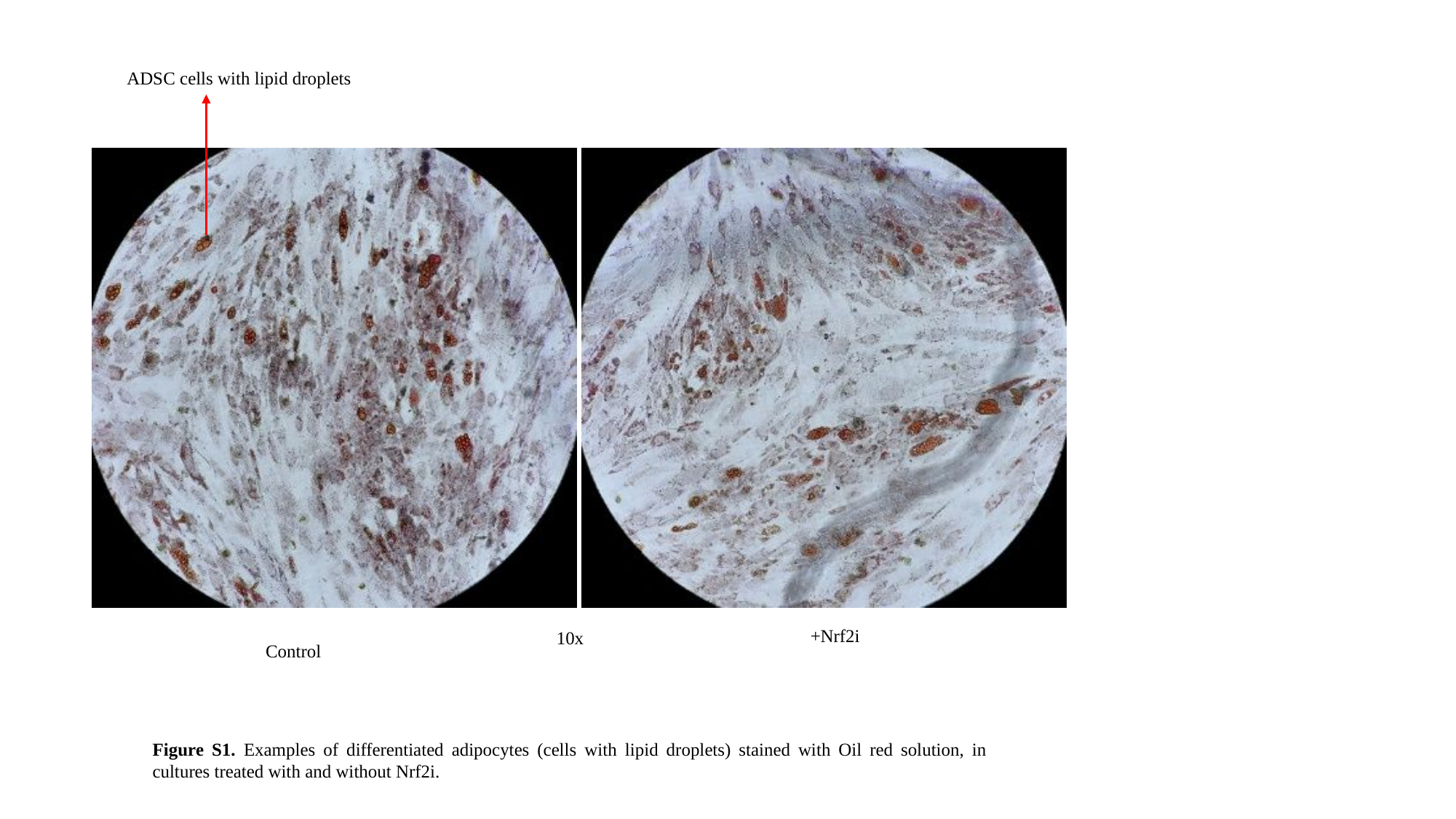

ADSC cells with lipid droplets
+Nrf2i
10x
Control
Figure S1. Examples of differentiated adipocytes (cells with lipid droplets) stained with Oil red solution, in cultures treated with and without Nrf2i.

## Slide 2
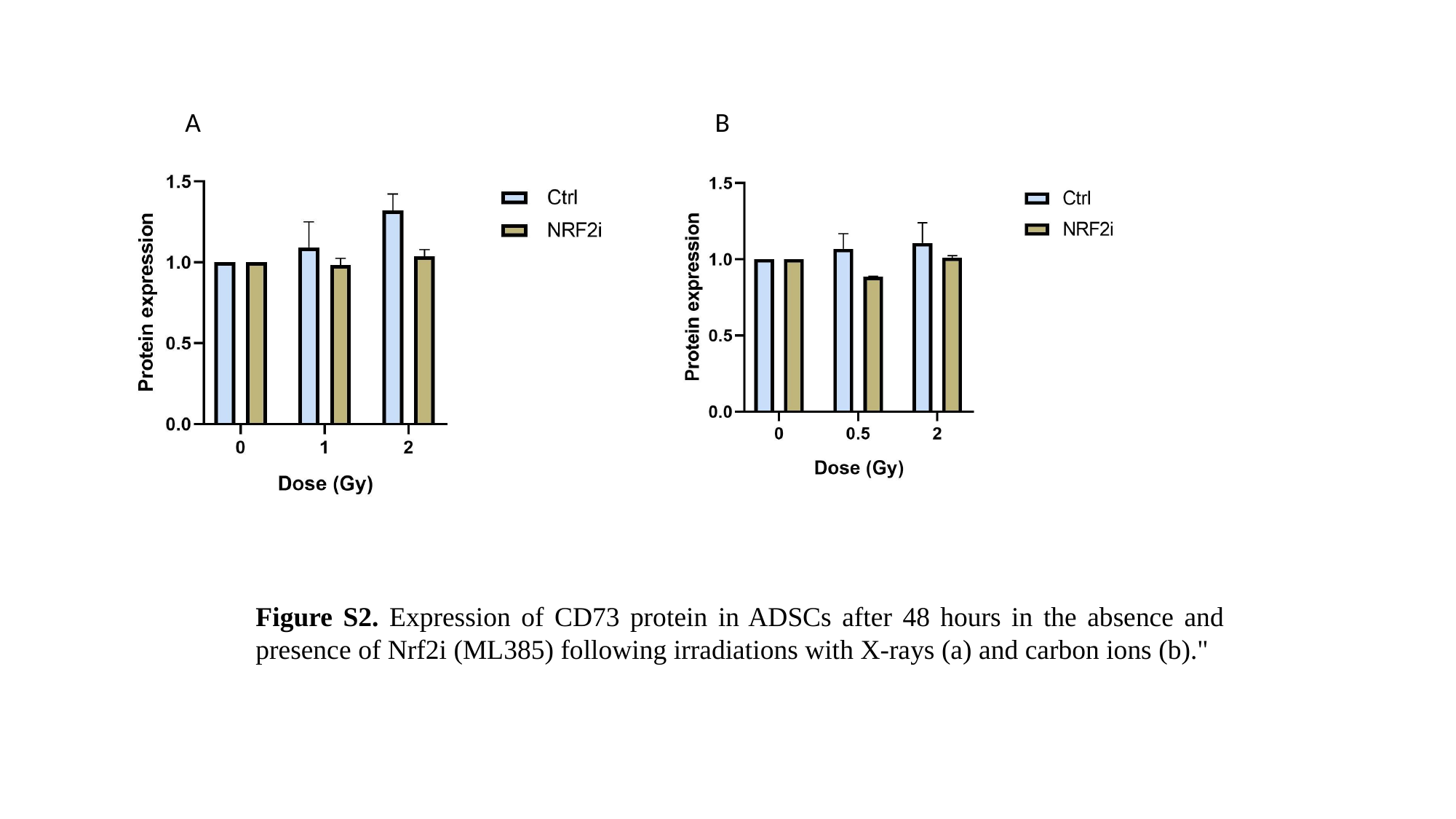

A
B
Figure S2. Expression of CD73 protein in ADSCs after 48 hours in the absence and presence of Nrf2i (ML385) following irradiations with X-rays (a) and carbon ions (b)."

## Slide 3
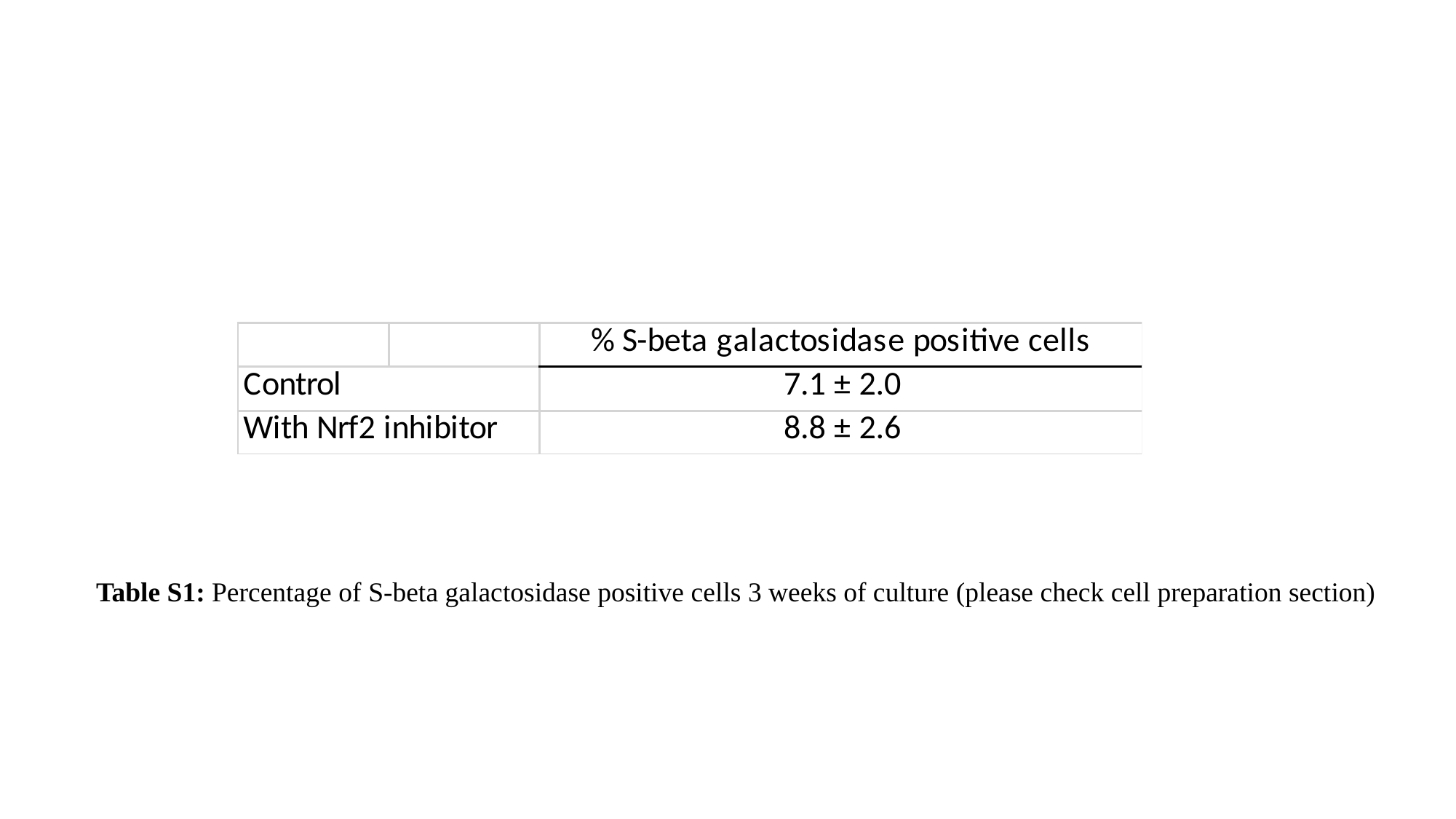

Table S1: Percentage of S-beta galactosidase positive cells 3 weeks of culture (please check cell preparation section)

## Slide 4
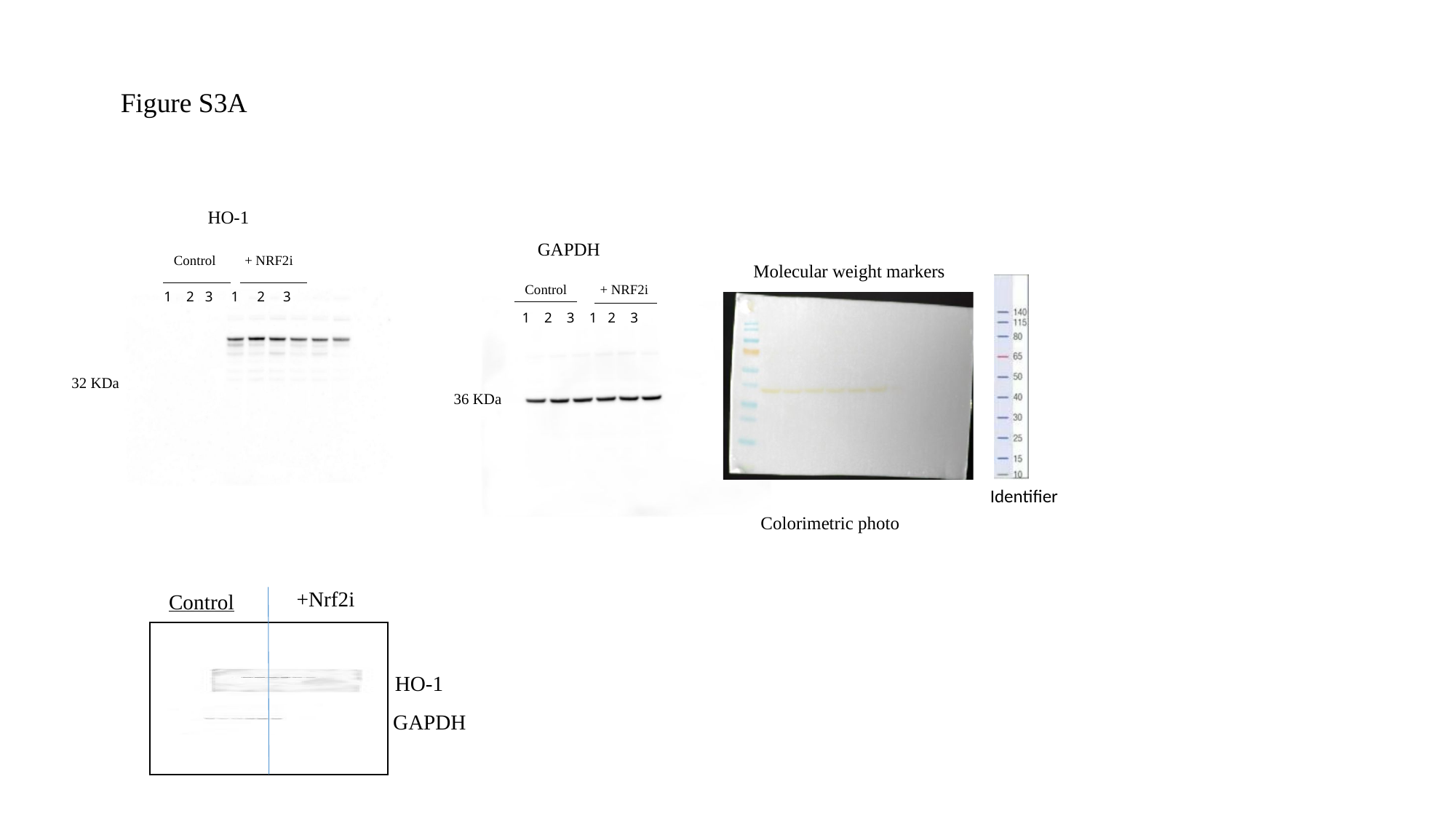

Figure S3A
HO-1
Control
+ NRF2i
 1 2 3 1 2 3
32 KDa
GAPDH
Molecular weight markers
+ NRF2i
Control
36 KDa
 1 2 3 1 2 3
Identifier
Colorimetric photo
+Nrf2i
Control
HO-1
GAPDH

## Slide 5
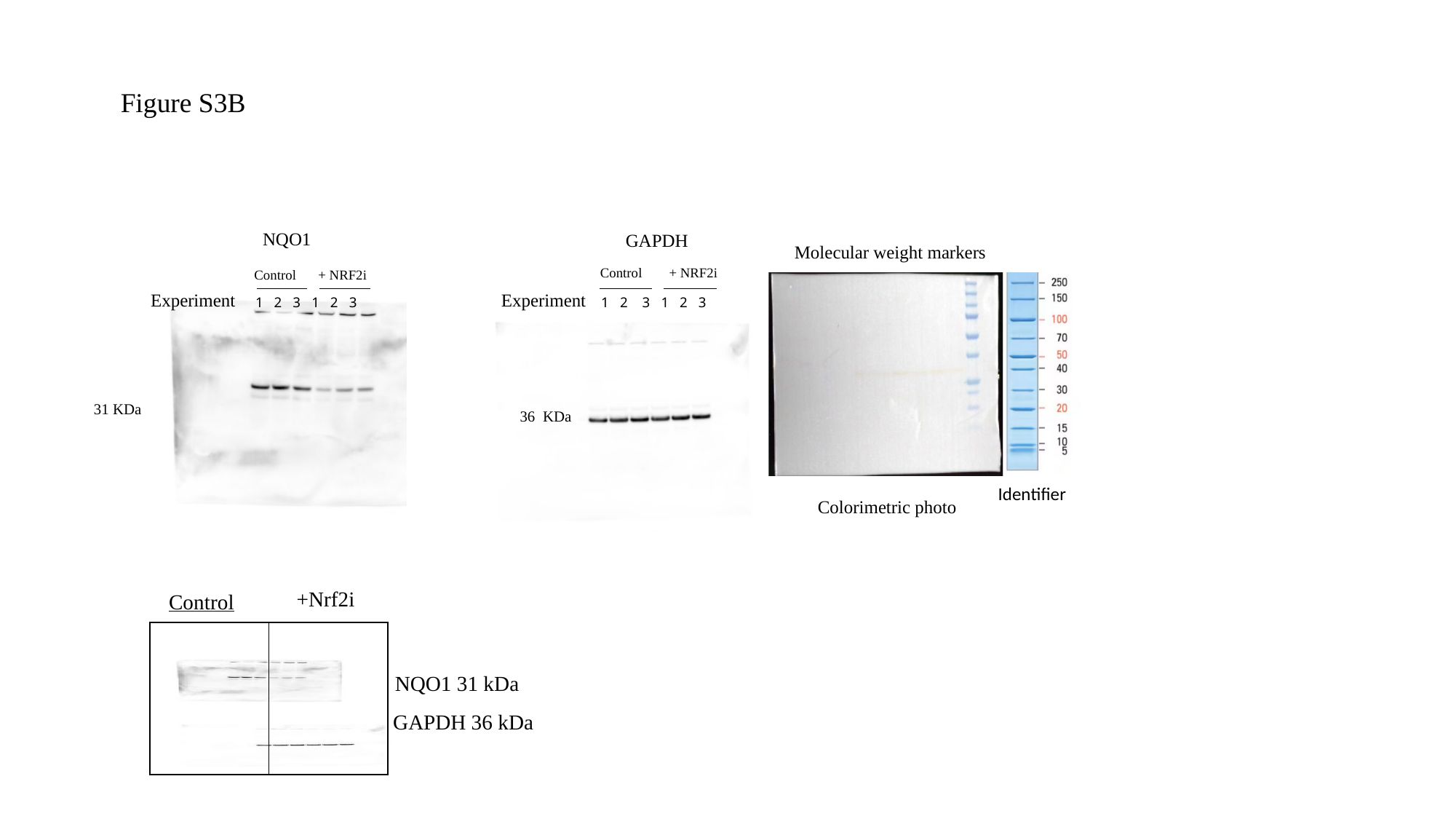

Figure S3B
NQO1
Molecular weight markers
GAPDH
Control
+ NRF2i
 1 2 3 1 2 3
36 KDa
Control
+ NRF2i
Experiment
Experiment
 1 2 3 1 2 3
31 KDa
Identifier
Colorimetric photo
+Nrf2i
Control
NQO1 31 kDa
GAPDH 36 kDa

## Slide 6
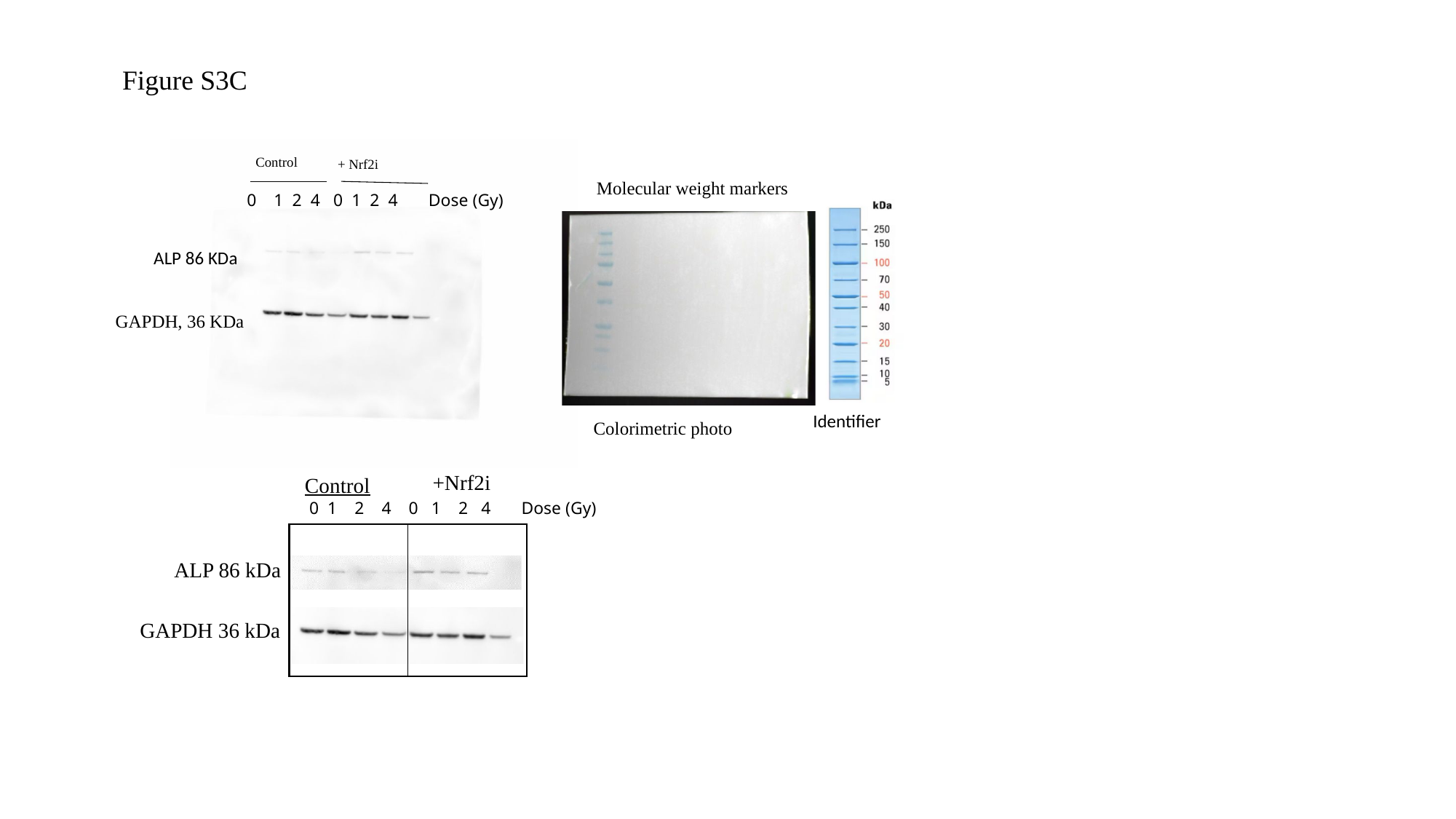

Figure S3C
Control
+ Nrf2i
Molecular weight markers
 0 1 2 4 0 1 2 4 Dose (Gy)
ALP 86 KDa
GAPDH, 36 KDa
Colorimetric photo
Identifier
+Nrf2i
Control
 0 1 2 4 0 1 2 4 Dose (Gy)
ALP 86 kDa
GAPDH 36 kDa

## Slide 7
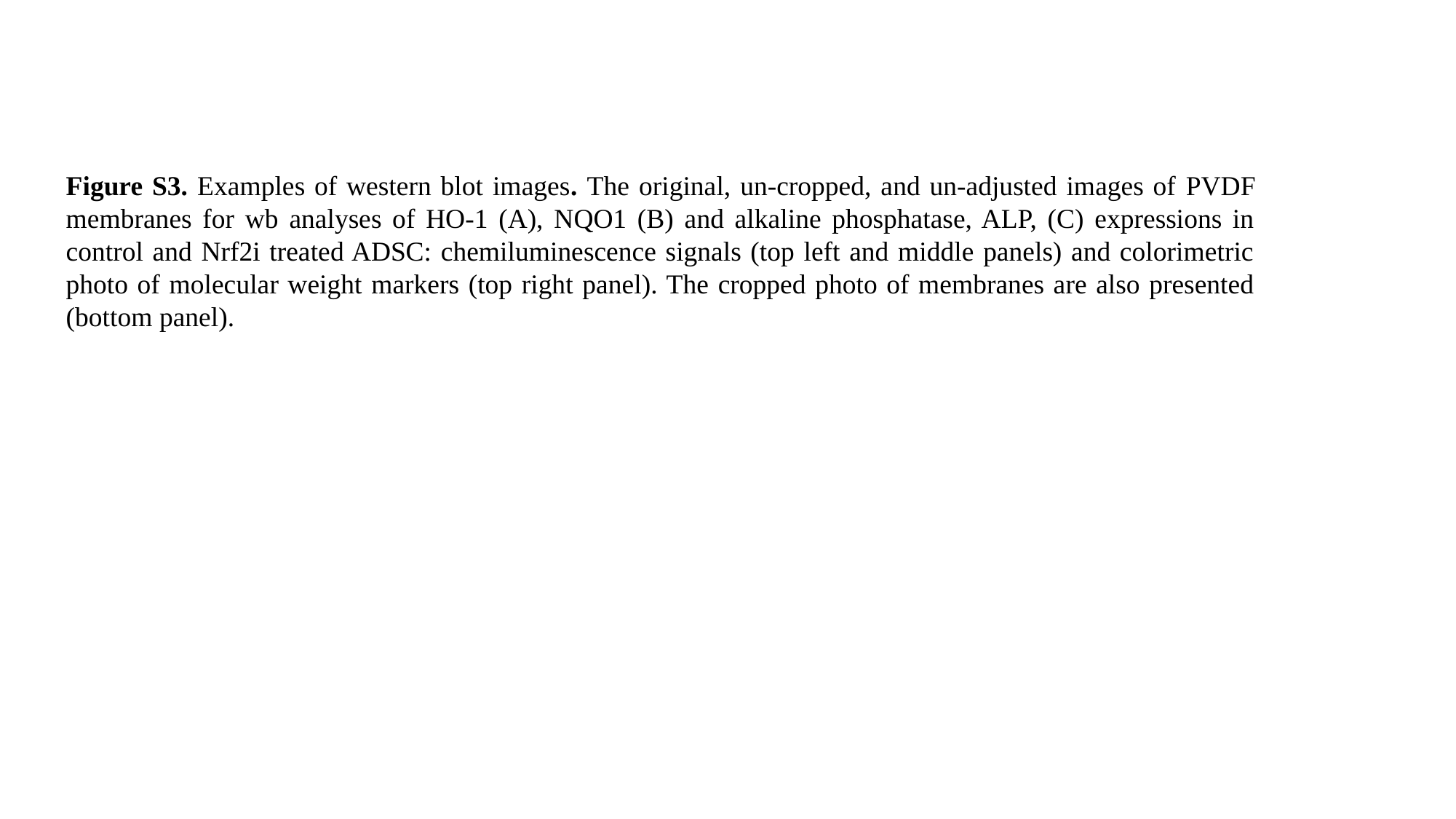

Figure S3. Examples of western blot images. The original, un-cropped, and un-adjusted images of PVDF membranes for wb analyses of HO-1 (A), NQO1 (B) and alkaline phosphatase, ALP, (C) expressions in control and Nrf2i treated ADSC: chemiluminescence signals (top left and middle panels) and colorimetric photo of molecular weight markers (top right panel). The cropped photo of membranes are also presented (bottom panel).
